# Introduction to the *RSC Advances* themed collection on *New insights into biomolecular systems from large-scale simulations*

**DOI:** 10.1039/d4ra90012j

**Published:** 2024-03-04

**Authors:** Megan L. O’Mara, Sarah Rauscher, Stacey D. Wetmore

**Affiliations:** a The University of Queensland, Australian Institute for Bioengineering and Nanotechnology Building 75, Cnr College Rd &, Cooper Rd St Lucia Queensland 4067 Australia; b University of Toronto Mississauga, Chemical and Physical Sciences 3359 Mississauga Rd N Mississauga Ontario L5L 1C6 Canada; c University of Toronto, Department of Physics 60 St. George St Toronto Ontario M5S 3H6 Canada; d Department of Chemistry 80 St. George St Toronto Ontario M5S 3H6 Canada; e University of Lethbridge, Department of Chemistry and Biochemistry 4401 University Drive West Lethbridge Alberta T1K 3M4 Canada stacey.wetmore@uleth.ca +1 (403) 329-2323

## Abstract

Megan O’Mara, Sarah Rauscher and Stacey Wetmore introduce the *RSC Advances* themed collection on *New insights into biomolecular systems from large-scale simulations*.
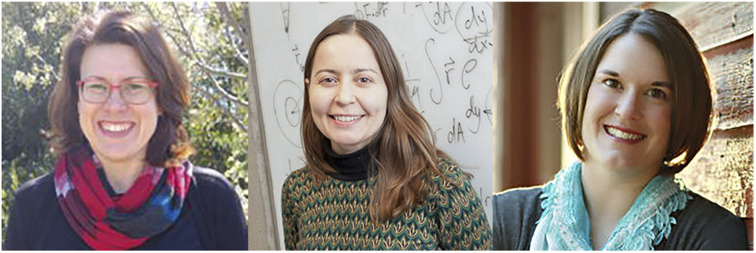

As a multidisciplinary area of research, computational biochemistry uses computer simulations to uncover molecular-level details across diverse sets of biomolecules. The past decade has been a particularly exciting turning point in the field, as modern computational resources are now sufficient to allow biological processes to be simulated at relevant timescales. New computational approaches have been developed that bridge the gap between simulations and experiments, allowing researchers to answer key questions about biomolecular dynamics and cellular function.

This themed collection in *RSC Advances* highlights recent applications of computer simulations to understand the structural and functional dynamics of biomolecular systems. Eighteen diverse contributions that use modelling to answer questions about the chemistry of biomolecules are included. The research highlighted uses a variety of simulation tools to study systems ranging from small nucleic acid components, to proteins and protein complexes, to large biomolecular complexes. For example, at the smallest scale, molecular dynamics (MD) simulations coupled with time-dependent density functional theory (TD-DFT) calculations and experimental data, provide critical information about nucleic acid base pairing and stacking interactions (https://doi.org/10.1039/D1RA00076D). At a much larger scale, simulations of a 1.2 million atom photosynthesis supercomplex reveal the molecular underpinnings of self-organization and regulation in plants and, more broadly, highlight the general principles for the assembly of macromolecular structures (https://doi.org/10.1039/D2RA08240C).

Computational methods have become extremely important tools for understanding the conformational dynamics, function, and biomolecular regulation, of proteins, highlighting aspects of clinical significance. For example, simulations have provided insight into the function of kinases that add phosphate groups to proteins, lipids, and nucleic acids, with hundreds of kinase-encoding genes being found in the human genome. MD simulations have shed light on the conformations of kinases necessary for regulation (https://doi.org/10.1039/D1RA01020D), while a combination of multi-conformation continuum electrostatics (MCCE) simulations, quantum mechanics/molecular mechanics (QM/MM) calculations, covalent docking, and MD simulations have been used to explain the high selectivity of inhibitors (https://doi.org/10.1039/D0RA01895C). Alternatively, simulations have proven useful for understanding conformational changes associated with protein mutations, which can facilitate small molecule inhibitor design (https://doi.org/10.1039/D2RA07472A) and the detailed characterization of conformational ensembles in solution and thus in cells (https://doi.org/10.1039/D2RA05324A). Simulations have also played a vital role in understanding protein interactions with nanomaterials, such as how wrinkles in nanomaterials induce protein unfolding (https://doi.org/10.1039/D2RA05489B).

Machine learning is emerging as a powerful new approach for understanding protein function. Indeed, data-driven machine learning models have impacted the field of biomolecular simulations and are now being used to shed light on protein folding, protein–ligand or protein–protein binding, and capturing conformational changes, among other cellular events. One contribution highlighted in this collection shows the ability to design and use machine learning models to predict the natural frequency spectrum of any protein without requiring a full atomistic model of its structure (https://doi.org/10.1039/C9RA04186A). The advent of machine learning algorithms to use collective variables to represent biomolecular dynamics has also been highlighted (https://doi.org/10.1039/D2RA03660F).

Moving to larger molecular scales, computer simulations can help us understand the chemistry of collagen, an essential component of the basement membrane that ensures proper structure and function of tissues. For example, classical atomistic MD and hybrid QM/MM techniques have provided critical insight into the formation and nature of the sulfilimine bond in collagen IV (https://doi.org/10.1039/D2RA02105F), while coarse-grained MD models of collagen IV networks in the cornea have highlighted the ability of simulations to capture phenomena of medical interest (https://doi.org/10.1039/D1RA07262E). Alternatively, membrane models can help scientists understand drug function and facilitate drug design. This collection highlights the ability of unbiased MD simulations and Markov state models to develop a permeability model for cyclic peptides through a lipid bilayer in hopes of extending the scope of druggable proteins (https://doi.org/10.1039/D1RA09025A), as well as how coarse-grained MD simulations can be used to understand the impact of hydrocortisone on a lung surfactant monolayer to enhance treatments for respiratory diseases (https://doi.org/10.1039/D2RA05268G).

The contributions in this themed collection in *RSC Advances* highlight the ability of computational models to address unexplored areas in biochemistry. We thank all authors for their contributions, and we hope that researchers working in the area of biomolecular simulations use these examples as inspiration to continue to apply simulations to advance our knowledge of cellular function from the perspective of the chemical sciences. In the future, scientists will continue to use simulations to study protein folding, intrinsically disordered proteins, protein aggregation, nucleic acid structure and function, membrane proteins, signalling molecules crossing membranes, and ligand interactions across length scales and time scales toward a deeper understanding of these chemical systems.

## Supplementary Material

